# Quarter-Annulus Si-Photodetector with Equal Inner and Outer Radii of Curvature for Reflective Photoplethysmography Sensors

**DOI:** 10.3390/bios14020109

**Published:** 2024-02-19

**Authors:** Yeeun Na, Chaehwan Kim, Keunhoi Kim, Tae Hyun Kim, Soo Hyun Kwon, Il-Suk Kang, Young Woo Jung, Tae Won Kim, Deok-Ho Cho, Jihwan An, Jong-Kwon Lee, Jongcheol Park

**Affiliations:** 1 Nano Convergence Technology Division, National Nano Fab Center, Yuseong-gu, Daejeon 34141, Republic of Korea; yeeun.na@nnfc.re.kr (Y.N.); chkim@nnfc.re.kr (C.K.); rmsghl12@nnfc.re.kr (K.K.); thk@nnfc.re.kr (T.H.K.); ksh@nnfc.re.kr (S.H.K.); iskang@nnfc.re.kr (I.-S.K.); 2 Sensor & Package Business Division, Partron Co., Ltd., Hwaseong-si 18449, Gyeonggi-do, Republic of Korea; ywjung@partron.co.kr (Y.W.J.); freektw@partron.co.kr (T.W.K.); 3 Research Department, Sigetronics Inc., Wanju-gun 55314, Jeollabuk-do, Republic of Korea; dhcho@sigetronics.com; 4 Department of Mechanical Engineering, Pohang University of Science and Technology, Pohang-si 37673, Gyeongsangbuk-do, Republic of Korea; jihwanan@postech.ac.kr; 5 Department of System Semiconductor Engineering, Cheongju University, Cheongju-si 28503, Chungcheongbuk-do, Republic of Korea

**Keywords:** photodiode, plasma dicing, photoplethysmography, non invasive, heart rate

## Abstract

Reflection-type photoplethysmography (PPG) pulse sensors used in wearable smart watches, true wireless stereo, etc., have been recently considered a key component for monitoring biological signals such as heart rate, SPO_3_, and blood pressure. Typically, the optical front end (OFE) of these PPG sensors is heterogeneously configured and packaged with light sources and receiver chips. In this paper, a novel quarter-annulus photodetector (NQAPD) with identical inner and outer radii of curvature has been developed using a plasma dicing process to realize a ring-type OFE receiver, which maximizes manufacturing efficiency and increases the detector collection area by 36.7% compared to the rectangular PD. The fabricated NQAPD exhibits a high quantum efficiency of over 90% in the wavelength of 500 nm to 740 nm and the highest quantum efficiency of 95% with a responsivity of 0.41 A/W at the wavelength of 530 nm. Also, the NQAPD is shown to increase the SNR of the PPG signal by 5 to 7.6 dB compared to the eight rectangular PDs. Thus, reflective PPG sensors constructed with NQAPD can be applied to various wearable devices requiring low power consumption, high performance, and cost-effectiveness.

## 1. Introduction

Reflectance-type photoplethysmography (PPG) sensors are regarded as pivotal components in wearable devices and are crucial for monitoring heart-related problems and facilitating continuous health tracking [1,2,3,4,5,6]. These PPG sensors are comprised of a light source to illuminate the skin tissue and a receiver to detect changes in light intensity associated with blood volume changes in the microvascular bed. In PPG sensors, the distance between the light-emitting devices and detectors is one of the most important design considerations because the optimized distance plays a pivotal role in enhancing the power efficiency and signal quality of PPG sensors by increasing their sensitivity to the reflected light. Thus, there is a need to develop a detector with an optimized structure that can efficiently collect light reflected from the skin around the light-emitting device. In this regard, Mendelson and Pujary proposed a PPG sensor with rectangular PDs placed in a ring-shaped annular position around a centrally positioned LED using a conventional off-chip package solution [7]. This ring configuration presents the potential of power saving for wearable devices. However, off-chip package solutions adopting separate LEDs and PDs have limitations in distance optimization due to the process of assembling individual chips on a package substrate and the use of a rectangular detector. Chen et al. introduced a PPG pulse sensor based on an integrated optoelectronic chip [8]. Here, the LED and PD were fabricated on the same epitaxial layer using GaN-on-sapphire wafers to reduce the distance between the emitting device and detector to several microns. The fabricated PPG optoelectronic chip can be directly attached to the skin by a flip-chip bonded on a flexible polydimethylsiloxane (PDMS) strip. Yan et al. also reported an integrated optoelectronics chip by adopting a ring structure for the detector to effectively collect the reflected light and improve performance [9]. This integrated PPG optoelectronic chip was fabricated on GaN-on-sapphire wafers and utilized two multiple-quantum-well (MQW) diodes for the LED and PD to achieve high sensitivity. However, this integrated PPG optoelectronic chip still has many issues to be solved for practical applications because of limitations in optimizing the performance of the LED and PD fabricated on the same epitaxial layer. Meanwhile, Lochner et al. proposed a transmission-type PPG sensor using two organic light-emitting diode (OLED) arrays and two organic photodiodes (OPDs) [10]. The organic optoelectronic devices were fabricated on a flexible plastic substrate using a solution process. The highly efficient polymer light-emitting diodes (PLEDs) and OPDs were designed for optoelectronic skins (oe-skins) with multiple functionalities on human skin. Yokota et al. also reported highly flexible PLEDs and OPDs with thin passivation layers for the realization of three-color (red, green, blue) PLEDs that can be worn on the skin and have similar performance to glass substrates [11]. In this configuration, half-ring-shaped green and red PLEDs encircled the centrally positioned OPD. Also, Lee et al. and Elsamnah et al. demonstrated comparable findings utilizing the organic PPG sensor comprised a ring-shaped OPD encircling a circular OLED [10,11]. These organic optoelectronics chips offer numerous advantages, including relatively low cost, monolithic integration, simple fabrication, and design flexibility. However, organic optoelectronic devices continue to encounter challenges in performance and reliability compared to conventional inorganic optoelectronic devices. To address these issues, we introduced a novel ring-shaped Si-photodetector to a PPG sensor using cost-effective Si fabrication and the advanced singulation process. Here, a plasma dicing process was applied to singulate a novel quarter-annulus photodetector (NQAPD) with equal inner and outer radii of curvature to maximize manufacturing efficiency and increase the detector collection area. Furthermore, we quantitatively investigated the performance of the fabricated PPG sensors adopting the NQAPDs or the conventional rectangular PDs to compare their feasibility and effectiveness as a reflection-type PPG sensor.

## 2. Materials and Methods

### 2.1. Design of the NQAPD

**Figure 1:**
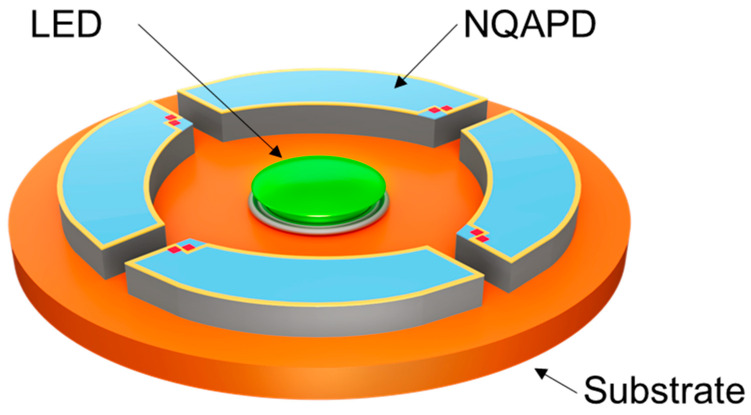
Schematic of the optical front-end with a ring-shaped receiving structure for the PPG sensor module using the proposed NQAPDs. Here, the receiving area of the PPG module was designed in a ring shape with an outer diameter of 9.2 mm and an inner diameter of 6 mm.

**Figure 2:**
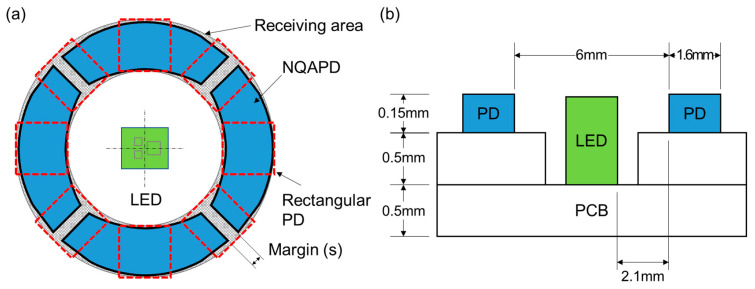
The dimensions of the optical front end with the ring-shaped receiving structure: (**a**) planar dimension and (**b**) cross-sectional dimension of the PPG sensor with the ring-shaped receiving structure using the proposed NQAPD.

As the light intensity detected by the photodetector relies on the fluctuating blood volume of the arterial blood vessels, a ring-shaped receiving structure was applied to enhance the light detection efficiency [9,12,13]. Previous works have considered multiple scattering and absorption of the light in the human skin for the design of PPG sensors. The scattering results from the diffusion of the emitted light from the LED in the human skin. Derraik et al. reported that dermal thickness varies between 1 and 2.5 mm depending on whether the user is obese or non-obese [14]. Therefore, the reflected light from the skin is distributed over the emitting surface that is several millimeters in diameter [12,13]. F. Elsamnah et al. presented an OPD area with an outer diameter of 8 mm and an inner diameter of 6 mm to obtain maximum irradiance. H. Lee et al. also reported ring-shaped OPDs with outer diameters of 7.6 mm and 9.2 mm for green and red wavelengths, respectively. Furthermore, Ajmal et al. determined that commercial products (Apple Watch series 5, Fitbit Versa 2 and Polar M600) have similar dimensions in their receiving areas using reverse engineering [15]. Based on this ring-shaped receiving structure, we proposed a PPG module using four NQAPDs as shown in Figure 1. The receiving area of the PPG module was designed in a ring shape with an outer diameter of 9.2 mm and an inner diameter of 6 mm to achieve the maximum irradiance on the PDs. Figure 2a,b show the planer and cross-sectional dimensions of the proposed PPG module adopting the proposed NQAPD. The distance between the LED package and the PD was 2.1 mm, and the LED package was embedded into the PCB substrate to diverge the emitting light efficiently by controlling the height of the emitting surface of the LED, as shown in Figure 2b. The light detection efficiency of PPG sensors depends on the sensing area of the PDs relative to the overall receiving area. For the ring-shaped receiving area (0.38 cm^2^) shown in Figure 2a, the effective sensing area for the PPG sensor module using the four NQAPDs or eight rectangular PDs (dimension of 1.6 × 1.6 mm^2^) has a coverage of 90.3% (0.34 cm^2^) or 53.6% (0.20 cm^2^), respectively. Thus, the proposed NQAPDs increase the coverage of the sensing area by approximately 36.7% and result in the improvement of the optical efficiency of PPG sensors.

In order to fabricate a quarter-annulus-shaped PD, we considered a 200 mm Si fabrication process. Here, implementing quarter-annular devices posed significant challenges, since traditional singulation processes involving blade dicing are primarily optimized for rectangular-shaped devices. Meanwhile, the laser ablation process provides greater flexibility in the singulation procedure but has the disadvantage of potentially causing contamination of the device surface during the singulation process [16,17]. For the NQAPD, the plasma dicing process was adopted for the singulation process of the curved dicing lane. The plasma dicing process consists of lithography for the etch mask and the anisotropic etching of the Si substrate. The lithography process defines the designated shape of devices and the width of the dicing lane by using photoresist. The anisotropic etching process typically uses Bosch deep reactive ion etching (DRIE) to achieve the high aspect ratio of the dicing lane. The DRIE process provides narrow etching of the Si substrate for singulation with minimal lateral etching. Therefore, this method allows design flexibility and minimizes surface contamination during the singulation process because of the dicing lanes defined by the lithography process and dry dicing using the DRIE process, respectively.

**Figure 3:**
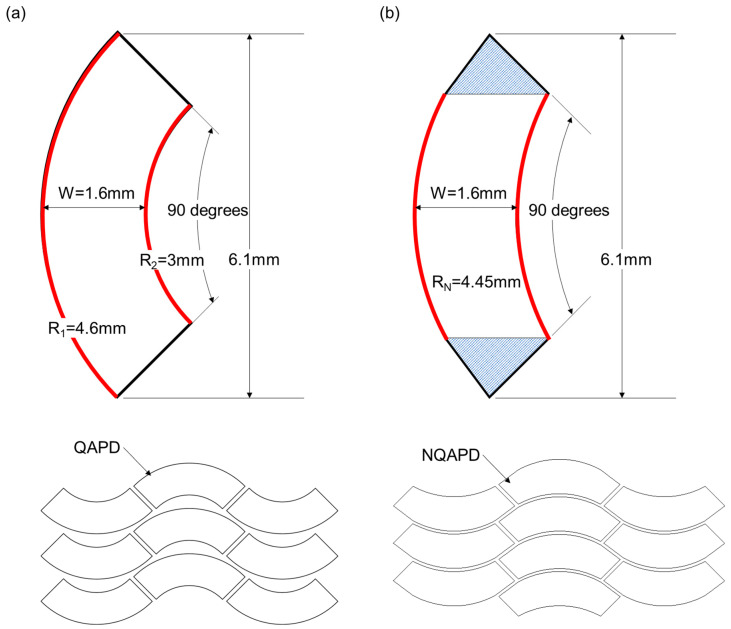
The design of photodiodes with a ring-shaped receiving structure: (**a**) the design of the QAPD with equal device width and zigzag layout on a wafer; (**b**) the design of the proposed NQAPD with equal inner and outer radii of curvature and efficient use of a wafer by zigzag arrangement.

Furthermore, the quarter annulus shape poses challenges in terms of fabrication efficiency. The conventional quarter annulus shape has different inner and outer radii of curvature for the quarter ring shape, as shown in Figure 3a. This difference of inner and outer radii causes considerable wastage of wafer area during the manufacturing process. To resolve this issue, the NQAPD was designed to have the same inner and outer radii of curvature, as shown in Figure 3b. This NQAPD was then structured as an arc shape, with the inner curvature forming a 90-degree angle and two triangular shapes (blue) at the ends of the arc. The distance between the two curvatures represents the maximum width of the NQAPD. The radii of NQAPD (RN) can be derived from the outer diameter (R_1_) of the sensing area and the assembly margin (S) of PDs for wire bonding and the die attach process, as shown in Figure 2a. Thus, the optimized radii of the NQAPD were rewritten as RN = R_1_ − S/2. This NQAPD can significantly maximize the fabrication efficiency compared to the typical QAPD and increase the detector collection area of PPG sensors compared to the rectangular PD. The proposed NQAPD has a uniform dicing lane as shown in Figure 3b. Here, the inner and outer dimensions of the NQAPD were defined by identical radii of 4.45 mm, separated by a distance of 1.6 mm. The radii of the curvature and width of the NQAPD were derived from the outer diameter (R_1_) of 9.2 mm for the sensing area and the margin (S) of 300 μm for the off-chip packaging method of wire bonding and die attaching, respectively. Thus, the NQAPD with an identical radius of 4.45 mm and a width of 1.6 mm had a device area of 8.62 mm^2^ and the typical QAPD with inner and outer radii of 4.6 and 3 mm had an area of 9.07 mm^2^. Although the size of the NQAPD has the disadvantage of a 5% smaller area than that of the typical QAPD, it ensures much higher manufacturing efficiency as shown in Figure 3 that anticipates larger yield and reduces fabrication costs. The overall size of the proposed NQAPD is 1.6 mm in width and 6.1 mm in height, comprising distinct segments including a guard ring, electrode part, and a light-receiving area.

### 2.2. Fabrication of the NQAPD

**Figure 4:**
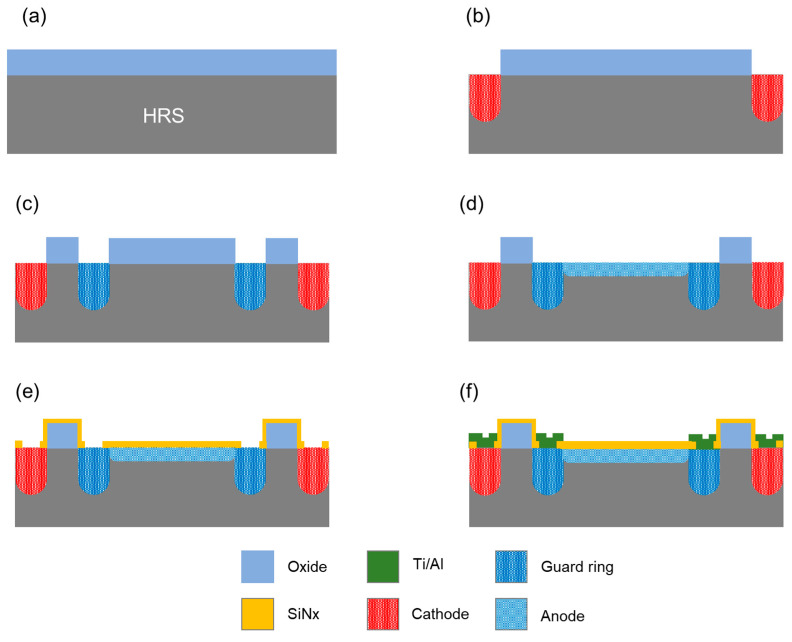
Fabrication process of NQAPD: (**a**) thermal oxidation of high-resistance n-type Si wafer for 8500 Å thick thermal SiO_2_ layer, (**b**) cathode implantation with Phosphorus at 80 keV and 5 × 10^15^ cm^−2^, (**c**) guard ring implantation with Boron at 80 keV and 5 × 10^14^ cm^−2^, (**d**) active implantation with Boron at 10 keV with a dose of 5 × 10^14^ cm^−2^, (**e**) AR layer deposition and via formation using 70 nm thick PECVD nitride layer, and (**f**) metallization using a typical lift-off process with 100 nm thick Ti layer and 1 μm thick Al layer.

The NQAPDs were fabricated with a PIN photodiode structure using a conventional 200 mm Si process as described in Figure 4. The process started with an 8-inch Si wafer doped with phosphorus ions, with a thickness of 725 μm and a high resistivity of 10 k ohm·cm. Firstly, an 8500 Å-thick thermal SiO_2_ layer was grown and used as a mask for ion implantation (using E1200 by Centrotherm, Blaubeuren, Germany) as shown in Figure 4a. The ion implantation process (using Phosphorus at 80 keV and 5 × 10^15^ cm^−2^) was conducted on the exposed Si area after removal of the oxide film for the cathode region (using EXELAN-HPT by Lam Research, Fremont, CA, US) as shown in Figure 4b. This was followed by an annealing process as a heat treatment method (at 1100 °C for 1 h). Then, an ion implantation process (utilizing Boron at 80 keV and 5 × 10^14^ cm^−2^) was applied to the exposed Si area after etching the silicon dioxide film as shown in Figure 4c. Subsequently, an annealing step was conducted at 1100 °C for 1 h to establish the guard ring structure, encircling the light-receiving area to increase the breakdown voltage [18]. The active area was specified by exposing the Si substrate through the process of patterning the SiO_2_ film within the guard ring structure. Then, the active region was formed by implanting Boron at 10 keV with a dose of 5 × 10^14^ cm^−2^, followed by rapid thermal processing (RTA) at 1000 °C for 10 s (using RTA200H-SP1 by NYMTECH, Jincheon-gun, Chungcheongbuk-do, Republic of Korea) as shown in Figure 4d. An anti-reflection (AR) layer with a thickness of 65 nm was deposited using PECVD SiN_x_ (through P-5000 by AMAT, Santa Clara, CA, USA) to enhance the quantum efficiency (QE) around green wavelength region as shown in Figure 4e. The metal films, consisting of a 100 nm thick Ti layer and a 1 μm thick Al layer, were deposited using an e-beam evaporator, and a standard lift-off process was used to create a metal pad on the etched contact region of the AR layer as shown in Figure 4f. The PIN photodiode structure for the proposed NQAPD is presented in Figure 4f.

**Figure 5:**
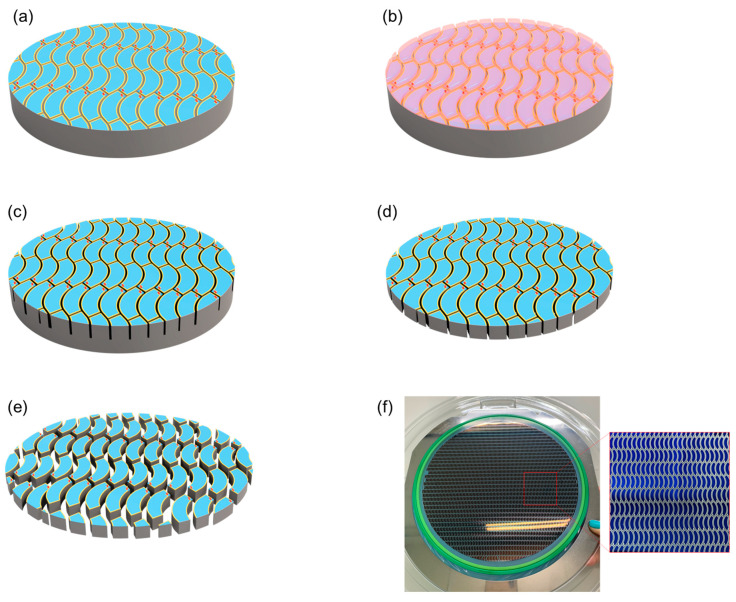
Fabrication procedure of the NQAPD using plasma dicing before the grinding process: (**a**) fabricated PDs using a 200 mm Si wafer process, (**b**) photolithography process to define the dicing lane of the NQAPD, (**c**) Si DRIE process for plasma half-cut, (**d**) thinning process for the singulation of the NQAPD, and (**e**) expanding process. (**f**) Photograph of the completed NQAPD in 8inch wafer state (left), enlarged NQAPD (right).

Next, the plasma dicing process was applied to fabricate the NQAPDs in a wafer state using the plasma dicing before grinding (PDBG) process as shown in Figure 5. This process involved two main steps: the first step was the plasma dicing method utilizing deep reactive ion etching (DRIE) on the front surface of the Si wafer substrate to achieve the desired depth. The second step involved the grinding process performed from the back side of the Si substrate for the purpose of singulation. The DRIE process within the dicing area, defined by the lithography process using an i-line contact aligner (EVG640 by EVG, St. Florian am Inn, Austria), was performed on the Si substrate with a depth of 170 μm by standard Bosch process (Neogen II by Gigalane, Hwaseong-si, Gyeonggi-do, Republic of Korea), as shown in Figure 5a,b. Then, the remaining photoresist was removed via the O_2_ plasma PR strip process (DAS-2000 by PSK, Hwaseong-si, Gyeonggi-do, Republic of Korea) as shown in Figure 5c, followed by applying a tape laminating process to the front side of the Si wafer for singulation. Finally, the NQAPD was then singulated by utilizing a backside grinding process to reduce the remaining thickness to 150 μm (using DGP8760 by DISCO, Ota-ku, Tokyo, Japan), as shown in Figure 5d. This grinding process included three stages conducted sequentially as follows: (1) the rough grinding process, (2) the fine grinding process for thinning the wafer to a designated thickness, and (3) the dry polishing process for stress relief of the grinded Si surface [19,20,21,22]. The singulated NQAPD on the 200 mm wafer using the PDBG was expanded post-process to avoid the collision during the pick-up of NQAPD with its neighbor as shown in Figure 5e. Figure 5f shows the fabricated NQAPDs placed on the dicing tape after the 200 mm Si PDBG process. The proposed NQAPD structure with an equal inner and outer radius of curvature provides efficient waver usage through the zigzag arrangement.

**Figure 6:**
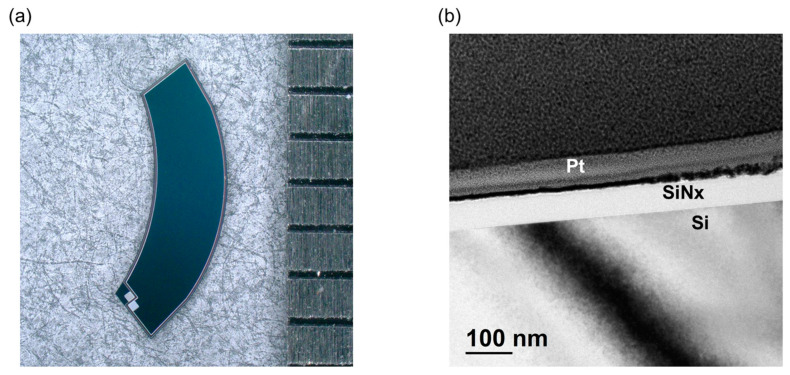
The fabricated NQAPD using plasma dicing before the grinding process: (**a**) photograph of fabricated NQAPDs on dicing tape using a 200 mm Si process and (**b**) the TEM image of cross-section of the fabricated NQAPD.

The fabricated NQAPDs with a length of 6156 μm, a width of 2365 μm, and a thickness of 150 μm were designed for use in the ring-shaped optical receiver of PPG sensors, as shown in Figure 6a. The SEM image of the AR layer formed by using PECVD SiN_x_ is presented in Figure 6b. The fabricated NQAPD was observed to have a 68 nm thick AR layer, slightly thicker than the 65 nm thickness corresponding to the SiN_x_ thin film with a refractive index of 2.035 at a wavelength of 530 nm [23]. These slightly thicker AR layers increase the minimum reflectance of the NQAPDs of about 20 nm toward the red. However, it is negligible in the overall performance of the NQAPD.

### 2.3. PPG Module

**Figure 7:**
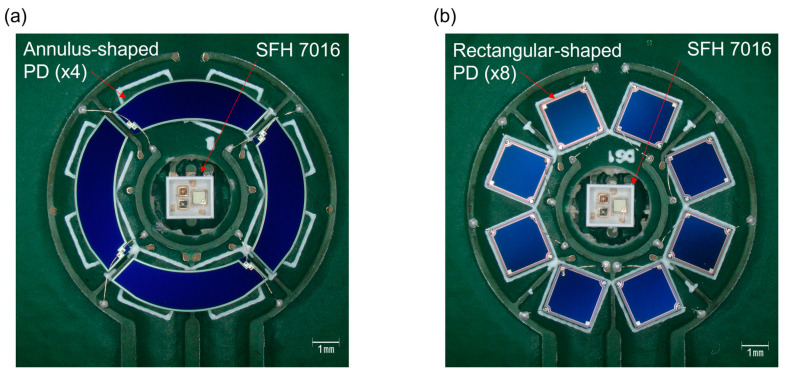
Photographs of the optical front end module of PPG sensors by integrating LEDs: (**a**) PPG sensor module using the fabricated four NQAPDs; (**b**) PPG sensor module using the conventional eight rectangular-shaped PDs.

The performance evaluation of the PPG modules was conducted to demonstrate the process capability and performance enhancement of the PPG sensors by the NQAPDs. For this purpose, two optical transceiver modules were interconnected with the photometric front end to analyze and characterize the PPG signals. Each PPG transceiver module comprised either four NQAPDs or eight rectangular PDs arranged at regular intervals in a configuration encircling a centrally positioned LED to ensure optimal signal reception and analysis. Here, multicolor LEDs catering to green, red, and near-infrared (NIR) light sources (SFH7016 from OSRAM, Munich, Germany) were used. The rectangular PDs for the transceiver module were fabricated with a similar PIN photodiode structure and an area of 2 × 2 mm^2^. The conventional off-chip packaging solution was utilized for the PD and LED assembly. The PDs were interconnected using wire bonding techniques to the package substrate to ensure a secure and reliable connection for data acquisition. The anode and cathode electrodes of each PD were interconnected in parallel for a single-channel input of the analog front end (AFE) chip. The photometric module incorporated the commercial front end chip (ADPD1081 by Analog Devices, Wilmington, MA, US) and was designed with reference to the evaluation kit (EVAL-ADPD1081Z-PPG by Analog Devices, Wilmington, MA, US), which guarantees a standardized and reliable assessment platform. Connecting the PPG module to the microcontroller board (EVAL-ADPDUCZ by Analog Devices, Wilmington, MA, US) enables seamless USB UART connectivity to the PC, allowing for efficient signal transmission and data acquisition. The Wavetool application by Analog Devices was utilized for the acquisition and analysis of the PPG signal data, offering a user-friendly and efficient platform for studying the captured data. Figure 7 illustrates the fabricated two optical transceiver modules for the PPG sensors. The ring-shaped optical receivers are demonstrated by using the fabricated four NQAPDs and the conventional eight rectangular PDs as shown in Figure 7a,b, respectively. The fabricated transceiver module was linked to the PPG front end chip module via a pin-header to control the integrated LEDs and acquire the PPG signal, using the microcontroller board connected to the PC. In order to protect the PDs and LED during the experiment, the fabricated transceiver modules were covered by using a 1 mm thick acrylic window and a 1 mm thick spacer to ensure the divergence of light emitted from the LED. 

## 3. Results and Discussion

### 3.1. Characterization of the NQAPD

**Figure 8:**
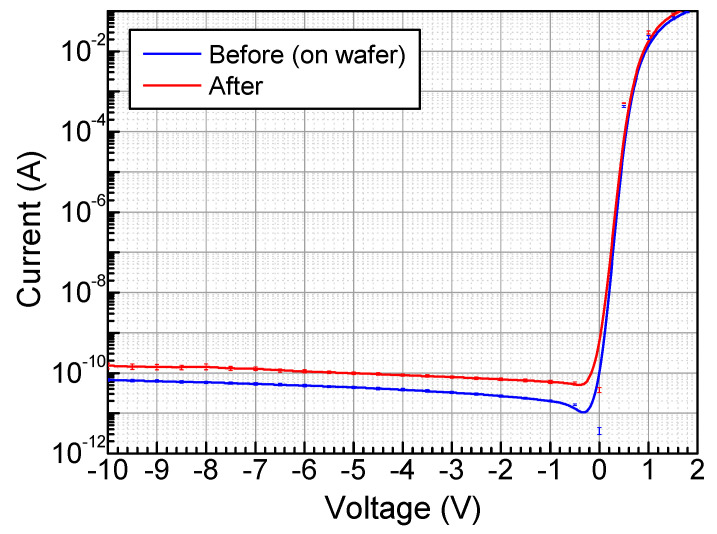
On-wafer measured I-V curves of the fabricated NQAPDs before and after the plasma dicing before the grinding process using a parameter analyzer (4200-SCS by Keithly, Beaverton, OR, US) under dark conditions (PAV200 by Formfactor, Livermore, CA, US).

Firstly, the performance of the fabricated NQAPDs was characterized to evaluate the PDBG process. Here, I-V measurements serve to ensure the suitability of a singulation method by assessing its impact on performance before and after the PDBG process. The I-V measurements before the PDBG process were performed at the wafer level, and the ones after the PDBG process were performed on the singulated NQAPD attached to the dicing tape. As an experimental setup, voltages ranging from −10 to 3 V were applied to the anode electrode at 0.5 V intervals, and the currents were measured using a parameter analyzer (4200-SCS by Keithly, Beaverton, OR, US). Figure 8 shows the I–V measurement results under dark conditions using the probe station (PAV200 by Formfactor, Livermore, CA, US). The dark currents before and after the PDBG for the fabricated NQAPD are both hundreds of pA, and in particular, it has a dark current of 99.2 ± 3.3 pA at −5 V. This indicates that the PDBG process maintains consistent performance with little effect on photodetector performance degradation.

**Figure 9:**
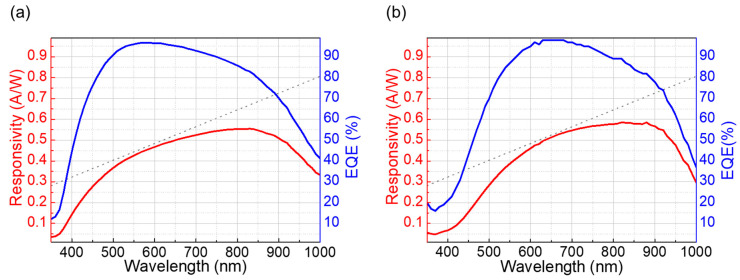
Measured SR and EQE of (**a**) the fabricated NQAPD and (**b**) the rectangular PD in a wavelength range from 350 to 1000 nm.

The spectral responsivity (SR) was measured in the visible-to-NIR wavelength (350 to 1000 nm) to characterize the optoelectronic performance of the fabricated NQAPDs. The SR measurement was conducted by the quantum efficiency measurement system (QUANTX-300 by Newport, Irvine, CA, US). A reverse bias of 5 V was applied for photocurrent mode operation, and the optical chopper frequency of 25 Hz was applied to be combined with a lock-in amplifier for the low noise measurement of the photocurrent by eliminating the external noise. The SR results of the fabricated NQAPD are presented in Figure 9a. The NQAPD reveals a high quantum efficiency of above 90% in a visible wavelength range from 500 nm to 740 nm. Especially, the responsivity at 530 nm wavelength is 0.41 A/W with an external quantum efficiency (EQE) of up to 95%. It is shown that the sensitivity to the green LED is noticeably improved by applying an anti-reflection thin film using PECVD SiN_x_ with a thickness of 68 nm. Furthermore, the responsivity of NQAPD at 670 nm wavelength was recorded as 0.51 A/W, indicating that it is suitable for operation with a red LED. Also, the maximum spectral response of 0.55 A/W is observed around the 800 nm wavelength.

The rectangular PD with a PIN structure was also fabricated and evaluated by using a 200 mm Si process for comparison with the NQAPD. The rectangular PDs exhibit EQE of above 90% in a visible wavelength range from 570 nm to 790 nm as shown in Figure 9b. The responsivity of the rectangular PD was observed to be 0.35A/W at a wavelength of 530 nm, which is comparably lower than the fabricated NQAPD. The maximum EQE of the rectangular PD is also seen to be 99% with the corresponding responsivity of 0.51 A/W at a 650 nm wavelength.

It is noted that reflectance-type PPG sensors typically utilize green LEDs for their adaptability to diverse human skin types and their reduced susceptibility to interference caused by thermal stress [3,24,25]. The multi-layered skin structure results in differences of the scattered light depending on the incidence wavelength on the skin. The short wavelength of green light is known to be easily absorbed by vessels and has a small penetration depth, while the longer wavelength of red or IR light can penetrate to deeper tissues [26]. Therefore, red or IR light has an advantage in picking up PPG signals from deep arteries in deeper tissues of human skin. However, the reflected and scattered light from the deeper tissues possesses complex information beyond the PPG signals. This is because the scattering and reflection of light incident on the skin mixes the pulsatile information of the arterioles and capillaries in the dermis and epidermis, respectively, and is affected by the noise of non-pulsatile media [27,28]. Y. Maeda et al. reported that green light has the advantage of a large component ratio (CR) in various environments [27]. A large CR directly associates with the SNR of PPG sensors for wearable devices. Thus, the fabricated NQAPD with high quantum efficiency in the green region is well suited for integration within PPG sensor modules in terms of performance improvement for wearable device applications.

### 3.2. Evaluation of the PPG Module

**Figure 10:**
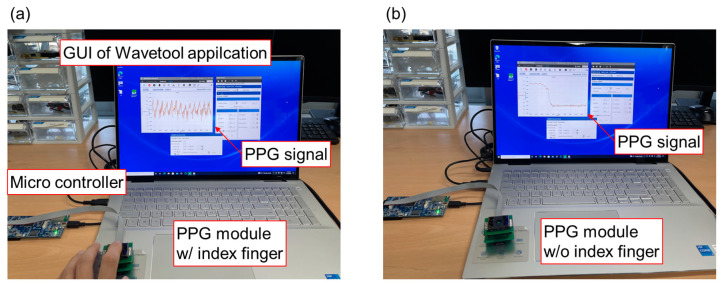
Photographs of the PPG sensor system operating (**a**) with and (**b**) without the index fingertip using the microcontroller board (EVAL-ADPDUCZ by Analog Devices, Wilmington, MA, US) connected to the PC.

**Figure 11:**
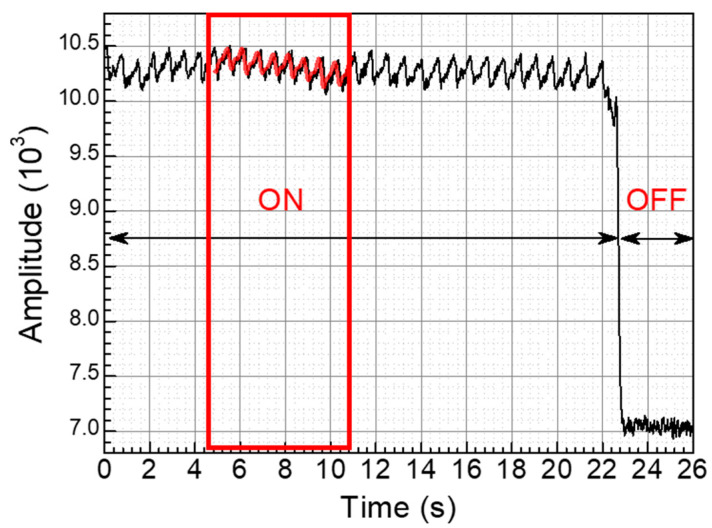
PPG waveforms acquired from the PPG sensor system using Wavetools with PPG evaluation kit.

The PPG signals were acquired using Wavetools (Analog Devices, Wilmington, MA, US) for the PPG evaluation kit provided by Analog Devices. The evaluation kit employed the front end chip ADPD1801Z, which was managed by several parameters to ensure optimal functionality. The PPG module was connected by using a 16-pin ribbon cable to the microcontroller board (EVAL-ADPDUCZ by Analog Devices, Wilmington, MA, US) for USB UART connection to the PC. Figure 10a shows the whole system for the PPG sensor module with the microcontroller board and the PC and also shows the photograph of the experimental setup for obtaining the PPG signal from an index fingertip. Figure 10 and Figure 11 show the experimental results of the PPG signal acquired from the PPG module at the tip of the index finger. The Wavetool application presents the obtained PPG signal from the microcontroller as shown in Figure 10a. Figure 10b shows the photograph of the experiment without a fingertip. The output of the sensor signal was significantly reduced, and the PPG signal was not detected. The raw data of the PPG signal saved in the PC was analyzed by using Origin Pro (Northampton, MA, US) as shown in Figure 11. The raw data represent the difference in amplitude of the PPG signal depending on whether the fingertip is placed on the PPG sensor or not, and the FFT-filtered signal smooths the PPG waveform.

**Figure 12:**
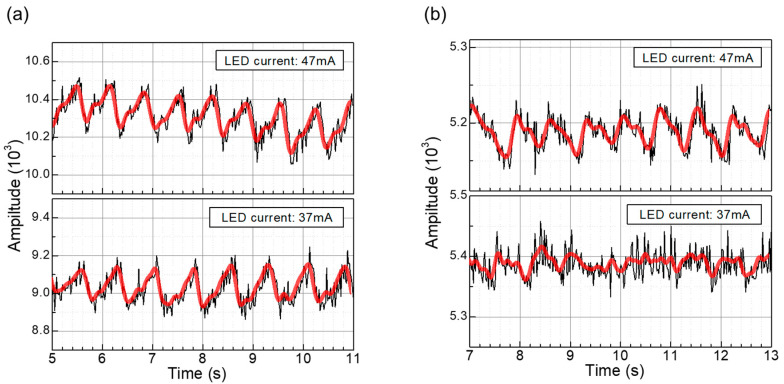
PPG waveforms acquired from the index fingertip through the PPG sensor with various LED drive currents: PPG waveform acquired from the optical front-end module using (**a**) the fabricated NQAPDs and (**b**) using the typical rectangular-shaped photodiodes. Black: the raw data from the PPG sensor modules by using Wavetool. Red: FFT filtered PPG waveforms using Origin Pro.

To compare the performance of the PPG modules utilizing the proposed NQAPDs and the conventional rectangular PDs, the sampling rate and the internal average were set as 100 Hz and 2, respectively. For evaluation purposes, the green LED associated with the SLOT B in the ADPD1081Z was adopted as the light source. The LED drivers were controlled with a fixed period and pulse of 19 μs and 1 μs, respectively. The PPG signals were obtained by optimizing the Transimpedance Amplifier (TIA) gain and LED currents. The TIA gains of the PPG modules utilizing the fabricated NQAPDs and the rectangular PDs were set at 25 k and 100 k, respectively. Additionally, the LED currents were controlled at 37 mA and 47 mA for the green LED, and the corresponding power consumption was 0.258 mW and 0.269 mW, respectively. Figure 12 shows the experimental results of the PPG waveform acquired from the PPG module at the tip of the index finger. The PPG signal acquired from the fingertip went through a smoothing process using an FFT filter, as shown in Figure 12. The demonstration using the proposed NQAPDs clearly presents the systolic and diastolic peak in the acquired waveform regardless of the drive current of the LED, as shown in Figure 12a. However, the acquired waveform from the front-end module using eight rectangular PDs has difficulty recognizing the cardiac cycle clearly at the low LED drive current as shown in Figure 12b.

**Figure 13:**
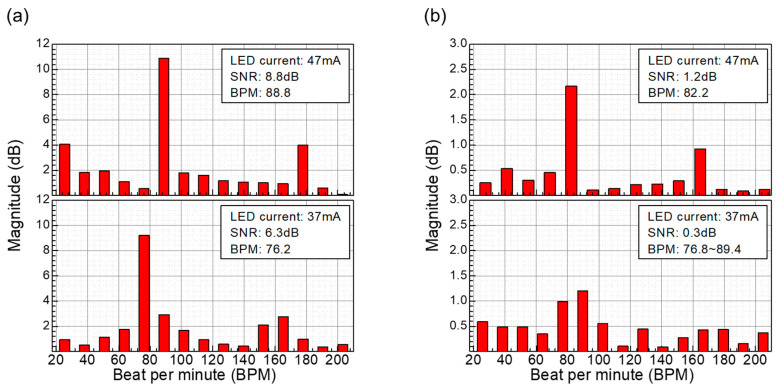
SNR per heart beat per minute acquired from the PPG waveform with smoothing processing using FFT filter: measured results from the optical front-end module (**a**) using the fabricated NQAPDs and (**b**) using the typical rectangular-shaped photodiodes.

These smoothed signals were then subjected to FFT analysis to extract heart rate signals, as shown in Figure 13. The resulting data show that the NQAPD-based PPG module achieves an SNR of 6.3 dB and 8.8 dB for green LED currents of 37 mA and 47 mA as shown in Figure 13a, respectively. Meanwhile, the rectangular PD-based PPG module reveals an SNR of 0.3 dB and 1.2 dB at the same LED currents as shown in Figure 13b. Thus, the proposed NQAPD-based module exhibits a larger SNR of 5 dB under a low LED current operation. The significance in the SNR of PPG sensor is associated with the accuracy of the signals. In wearable devices such as smart watches, TWS, smart rings, etc., battery lifetime is the one of the most importance features. For PPG sensors, the LED driver consumes the most power for the operation of PPG sensors because it requires several tens of mA of current to obtain accurate PPG signals. In order to reduce the power consumption, the pulsed LED light is adopted by controlling the duty-cycle ratio of turning on the LED in AFE [29,30,31,32,33]. The proposed NQAPD provides a physically increased detection area for the low-current operation of an LED driver for the PPG signals by using a ring-shaped detector [8,9,11,12]. These results show a significant contribution to enhancing the power efficiency of wearable devices.

## 4. Conclusions

We developed the NQAPDs as novel ring-shaped receivers for PPG sensors. These NQAPDs were designed in such a way to have equal inner and outer radii of curvature to maximize the fabrication efficiency for large-area Si process. It was observed that the PDBG process presented the capability of design flexibility and low contamination for optoelectronic devices. The PPG sensor adopting four NQAPDs was demonstrated by using a conventional off-chip packaging solution. The performance of the fabricated NQAPD showed a high quantum efficiency of above 90% in the wavelength from 500 nm to 740 nm. Especially, the responsivities of 0.41 A/W and 0.51 A/W at 530 nm and 670 nm wavelengths, respectively, reveal the suitability for operation with green and red LEDs for PPG sensor modules. Also, the fabricated NQAPD exhibited a larger 5 dB SNR of the PPG sensor module compared to the PPG sensor adopting rectangular-shaped PDs at low LED currents. Therefore, the NQAPD fabricated using a large-area Si process can be applied to diverse wearable devices that detect monitoring biological signals due to its low power consumption, high performance, and low-cost features.

## Data Availability

Data underlying the results presented in this paper are available from the corresponding author upon reasonable request.
